# History of anemia and long-term mortality due to infection: a cohort study with 12 years follow-up in South Korea

**DOI:** 10.1186/s12879-021-06377-0

**Published:** 2021-07-11

**Authors:** Tak Kyu Oh, Kyung-Ho Song, In-Ae Song

**Affiliations:** 1 Department of Anesthesiology and Pain Medicine, Seoul National University Bundang Hospital, Gumi-ro, 173, Beon-gil, Bundang-gu, Seongnam, 13620 South Korea; 2 Department of Internal Medicine, Seoul National University Bundang Hospital, Gumi-ro, 173, Beon-gil, Bundang-gu, Seongnam, 13620 South Korea

**Keywords:** Hematology, Infectious diseases, Public health, Long-term mortality, Anemia

## Abstract

**Background:**

Anemia, which is a condition with reduced healthy red blood cells, is reported to be closely related to the development of infectious diseases. We aimed to investigate the association between history of anemia and 12-year mortality rate due to infections, and compare it with that among non-anemic individuals.

**Methods:**

Data from the National Health Insurance Service Health Screening Cohort were used in this population-based cohort study. Adults who underwent standardized medical examination between and 2002–2003 were included, and the mortality rate due to infection between 2004 and 2015 was analyzed. Individuals were considered to have a history of anemia if the serum hemoglobin level in 2002–2003 was < 12 g/dL for women and < 13 g/dL for men. The severity of anemia at that time was categorized as mild (12 g/dL > hemoglobin ≥11 g/dL in women and 13 g/dL > hemoglobin ≥11 g/dL in men), moderate (hemoglobin 8–10.9 g/dL), or severe (hemoglobin < 8 g/dL). Propensity score (PS) matching and Cox regression analysis were used as statistical methods.

**Results:**

Overall, 512,905 individuals were included in this study. The mean age of the participants was 54.5 years old (range: 40–98), and 49,042 (9.6%) individuals were classified in the anemic group, which comprised of 36,383 (7.1%), 11,787 (2.3%), and 872 (0.2%) participants in the mild, moderate, and severe sub-groups, respectively. After PS matching, 49,039 individuals in each group were included in the analysis. The risk of mortality due to infection in the anemic group was 1.77-fold higher (hazard ratio [HR]: 1.77, 95% confidence interval [CI]: 1.52–2.60; *P* < 0.001) than that in the non-anemic group. In the subgroup analysis, the mild and moderate anemia groups had 1.38-fold (HR: 1.38, 95% CI: 1.23 to 1.55; *P* < 0.001) and 2.02-fold (HR: 2.02, 95% CI: 1.62 to 2.50; *P* < 0.001) risk of mortality due to infection compared to that of the non-anemic group, respectively. The severe anemia group did not have a significantly different risk of mortality due to infection (*P* = 0.448).

**Conclusions:**

History of anemia was associated with increased mortality rate due to infection at 12-year follow-up.

**Supplementary Information:**

The online version contains supplementary material available at 10.1186/s12879-021-06377-0.

## Background

Infection is defined as the entrance and development of an infectious agent in a human, regardless of whether it develops into an infectious disease [1]. As a major health issue, infectious disease is one of the most important causes of mortality and morbidity in human history [2]. Mortality and morbidity due to infectious diseases are common despite advances in medicine. In South Korea, the age-standardized mortality due to infection has increased from 16.5/100,000 in 1996 to 44.6/100,000 in 2015 [3]. Although there has been an improvement in infant mortality rates due to infectious diseases, the mortality rate among older adults has increased, and death rates from infectious diseases are still important public health issues, which need to be addressed in South Korea [3]. Currently, the world population suffers from coronavirus disease-2019 (COVID-19) pandemic as a global public health crisis [4].

Anemia is a chronic disease in which a person’s hemoglobin levels are lower than required to meet their physiological needs, and affects roughly one-third of the global population [5].

In South Korea, the total prevalence of anemia was 6.0%, and the prevalence of severe anemia was 0.92% [6]. There has been some evidences that anemia is closely related to the development of infectious disease [7]. Since iron is an immunomodulating nutrient that can regulate humoral and cellular immunity, iron deficiency has been identified as the most common cause of anemia [8]. Thus, immunogenic mechanisms such as cytokine activity, humoral, cell-mediated, and non-specific immunity have been shown to be negatively influenced in patients with iron deficiency anemia (IDA) [9]. Considering that infection increases the demand for iron for immunoreactions [9, 10], patients with IDA may be more susceptible to infectious disease-related mortality; some previous studies reported that anemia was a risk factor for lower respiratory infections [11, 12] and subclinical infections in children [13]. Moreover, deficiency of micronutrients, such as vitamin A, folate, and vitamin B12 in addition to iron deficiency is the most common cause of anemia [14, 15]. As the micronutrient deficiencies are known to play a major role in the innate and adaptive immune responses to infections such as COVID-19 [16], the comorbid anemia which may be caused by micronutrient deficiencies could also be associated with increased risk of infection. However, the relationship between history of anemia and long-term mortality rate due to infection has not been investigated yet.

Therefore, this study aimed to investigate the hypothesis that history of anemia might be an independent risk factor for higher mortality rate due to infection.

## Methods

### Study design and ethical statements

This study involved human participants, and all procedures were conducted in accordance with the guidance provided by the relevant ethics boards. The Institutional Review Board (IRB) of Seoul National University Bundang Hospital (IRB approval number: X-1911-579-902) and the Health Insurance Review and Assessment Service (NHIS-2020-2-067) approved the study protocol. Informed consent was waived by IRB of Seoul National University Bundang Hospital, because data analyses were performed retrospectively using anonymized health records derived from the South Korean NHIS database. Data were extracted by an independent medical record technician at the NHIS center who was unaffiliated with this study.

### Data source: NHIS-HEALS database and study population

The NHIS-National Health Screening Cohort (NHIS-HEALS) was used in this study [17]. As the sole public insurance system in South Korea, the NHIS collects information regarding demographics; socioeconomic status; diagnosis of diseases according to the International Classification of Diseases, tenth revision (ICD)-10 codes; and treatment for the diseases. Subscribers to the NHIS who are ≥40 years old are recommended to receive standardized medical examination every 2 years [18]. Using the results of the standardized medical examination, the NHIS constructed the NHIS-HEALS database for medical research. The cohort comprised 514,795 individuals who underwent standardized medical examination between 2002 and 2003, and were followed up until 2015. The database contains information regarding body mass index (BMI), laboratory test results including hemoglobin, and questionnaires on lifestyle (exercise, alcohol consumption, and smoking). We included individuals who underwent a standardized medical examination during 2002–2003 for this study. However, data of individuals who died between 2002 and 2003, or had missing data on hemoglobin were excluded from the analysis.

### Exposure: history of anemia

All individuals were divided into two groups: the anemic (who had a history of anemia) and non-anemic groups. Individuals who had hemoglobin levels < 12 g/dL for women and < 13 g/dL for men, during 2003–2003, were considered to have anemia based on the World Health Organization (WHO) criteria. The severity of anemia at that time was categorized as mild (12 g/dL > hemoglobin ≥11 g/dL in women and 13 g/dL > hemoglobin ≥11 g/dL in men), moderate (hemoglobin 8–10.9 g/dL), or severe (hemoglobin < 8 g/dL), using the WHO criteria [19]. Serum hemoglobin concentration was measured using the cyanmethemoglobin method. If the hemoglobin level of individuals was measured twice between 2002 and 2003, the hemoglobin level in 2003 was used to diagnose and classify anemia.

### Study endpoint: mortality due to infection

In this study, mortality due to a primary infection was considered as the study endpoint. The NHIS database provided data on the death date and main cause of death for all individuals. Mortality rate due to infection was evaluated for a period of 12-years, from January 1, 2004 to December 31, 2015. The specific diagnoses for mortality due to infection are presented as ICD-10 codes in Table S1.

### Covariates

The following variables were collected as covariates for this study: demographic information (age, sex, and BMI), socioeconomic status related information (residence and annual income level), comorbidity related information (underlying disability and Charlson comorbidity index), and lifestyle information (smoking status, alcohol consumption, and exercise frequency). Residence was divided into three groups (Seoul, other metropolitan cities, and other areas), and BMI was categorized into four groups (below 18.5, 18.5–24.9, 25.0–29.9, and > 30 kg/m^2^). The national income level was registered in the NHIS database to determine the insurance premium of all individuals. Annual income level was divided into five groups using quintile ratio (1st: 0–20% [lowest], 2nd: 20–40%, 3rd: 40–60%, 4th: 60–80%, and 5th 80–100% [highest]), and underlying disability was divided into two groups (mild to moderate, and severe). In South Korea, all physical disabilities should be registered in the NHIS to receive various benefits, and are divided into six levels considering their severity. Thus, in this study, disabilities in the 1st (most severe) to 3rd levels were classified in the severe disability group, while those in the 4th to 6th (most mild) levels were classified in the mild to moderate disability group. Smoking status was divided into four groups (never smoked, previous smoker, current smoker, and unknown [no-response group]), and alcohol consumption was divided into six groups (does not drink, 2–3 drinks per month, 1–2 drinks per week, 3–4 drinks per week, drink almost every day, and unknown [no-response group]). Exercise frequency was divided into six groups (no exercise, exercise 1–2 times per week, 3–4 times per week, 5–6 times per week, exercise almost every day, and unknown [no-response group]). The Charlson comorbidity index was calculated using registered ICD-10 codes from to 2002–2003, as shown in Table S2 [20].

### Statistical analysis

The clinico-epidemiological characteristics of the individuals are presented as mean values with standard deviations for continuous variables and numbers with percentages for categorical variables. First, we performed 1:1 propensity score (PS) matching between the anemic group (those with a history of anemia) and non-anemic group to reduce confounders [21]. For this PS-matching, the nearest neighbor method was used without replacement with a caliper of 0.25. All covariates were included in the PS model, and logistic regression analysis was performed to calculate the PSs. The absolute value of the standardized mean difference (ASD) was used to evaluate the balance between the groups before and after PS-matching. The ASD was set at < 0.1 to confirm adequate balance between the groups. After confirming adequate balance, we performed Cox proportional hazards regression analysis for mortality rate due to infection in the PS-matched cohort. In this time to event analysis, death due to infection was set as the event, and survival time from January 1, 2004 to death date was set as the duration. As a first sensitivity analysis, we investigated the association between the anemic group and mortality due to infection during 2005–2015, and not 2004–2015, in the PS-matched cohort to avoid reverse causation bias because there was a short latency time between history of anemia and mortality due to infection in 2004 [22].

As a second sensitivity analysis, we constructed a multivariable Cox regression model for mortality due to infection using the entire cohort to determine: (1) whether the results obtained from the PS-matched cohort were generalizable to the entire cohort, and (2) the risk of mortality due to infection in the anemic group with other important covariates in context, and not in isolation. All covariates were included in the multivariate Cox model for adjustment. Using multivariable Cox regression modeling, we performed subgroup analyses to investigate whether mild, moderate, and severe anemia in the past, were associated with mortality due to infection compared to the non-anemic group. In addition, considering sex is associated with development of anemia [23], we performed subgroup analysis stratified by sex to examine the impact of sex on the association between history of anemia and mortality due to infection. We confirmed that there was no multicollinearity in all multivariable models involving the entire cohort, with a variance inflation factor of < 2.0. The results of the Cox regression are presented as hazard ratios (HRs) with 95% confidence intervals (CIs). C-statistics were used to identify the C-index of the multivariable Cox regression model. All statistical analyses were performed using R software (version 4.0.3 with R packages, the R Project for Statistical Computing, Vienna, Austria). *P* < 0.05 was considered statistically significant.

## Results

### Study population

**Fig. 1:**
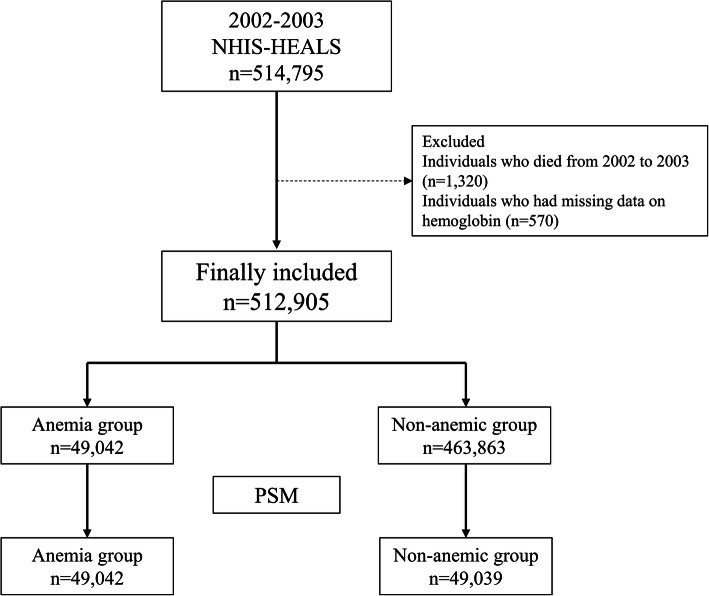
Flow chart depicting participant selection NHIS-HEALS, National Health Insurance Service-National Health Screening Cohort; PSM, propensity score matching.

In the NHIS-HEALS data, a total of 514,795 individuals received standardized medical examination from 2002 to 2003. Among them, 1320 individuals were excluded due to death during 2002–2003, and 570 individuals were excluded due to missing data regarding hemoglobin level. Thus, a total of 512,905 individuals were included in this study. Among them, 49,042 individuals were classified as the anemic group (9.6%) using serum hemoglobin level, and 463,863 individuals (90.4%) were classified as the non-anemic group. After PS-matching, a total of 98,078 individuals (49,039 in both groups) were included in the analysis (Fig.1). The results of the comparison of clinico-epidemiological characteristics between the anemic and non-anemic groups before and after PS-matching are presented in Table 1. All ASDs between the two groups were below 0.1 after PS-matching, showing adequate balance through PS-matching.

**Table 1:** Comparision of clinico-epidemiological characteristics between anemia group and non-anemic group before and after PS matching

Variable	Total Cohort (*n* = 512,905)		ASD	PS-matched cohort (*n* = 98,078)		ASD
	Anemia group *n* = 49,042	Non-anemic group *n* = 463,863		Anemia group *n* = 49,039	Non-anemic group *n* = 49,039	
Age, year	54.9 (10.8)	53.5 (9.5)	0.134	54.9 (10.8)	54.7 (9.8)	0.017
Sex, male	12,778 (26.1)	265,068 (57.1)	0.708	12,778 (26.1)	12,458 (25.4)	0.015
Residence						
Seoul (Capital city)	8214 (16.7)	79,819 (17.2)		8213 (16.7)	8280 (16.9)	
Other metropolitan city	12,838 (26.2)	127,430 (27.5)	0.035	12,838 (26.2)	13,065 (26.6)	0.011
Other area	27,990 (57.1)	256,614 (55.3)	0.125	27,988 (57.1)	27,694 (56.5)	0.012
Body mass index, kg/m^2^						
18.5–24.9 (normal)	34,312 (70.0)	286,642 (61.8)		34,311 (70.0)	33,639 (68.6)	
Below 18.5 (Underweight) 25.0–29.9 (Overweight)	2302 (4.7)	9561 (2.1)	0.125	2300 (4.7)	2016 (4.1)	0.027
	11,384 (23.2)	153,504 (33.1)	0.234	11,384 (23.2)	12,229 (24.9)	0.041
Above 30.0 (Obese)	982 (2.0)	13,741 (3.0)	0.069	982 (2.0)	1094 (2.2)	0.016
Unknwon	62 (0.1)	415 (0.1)	0.010	62 (0.1)	61 (0.1)	< 0.001
Annual income level						
0–20%	9698 (19.8)	70,879 (15.3)		9698 (19.8)	9101 (18.6)	
20–40%	7819 (15.9)	62,101 (13.4)	0.070	7818 (15.9)	7515 (15.3)	0.017
40–60%	7811 (15.9)	73,151 (15.8)	0.004	7811 (15.9)	7969 (16.3)	0.009
60–80%	9492 (19.4)	98,521 (21.2)	0.048	9492 (19.4)	9690 (19.8)	0.010
80–100%	14,222 (29.0)	159,211 (34.3)	0.117	14,220 (29.0)	14,764 (30.1)	0.024
Underlying disability						
Mild to moderate	228 (0.5)	1824 (0.4)	0.011	227 (0.5)	216 (0.4)	0.003
Severe	196 (0.4)	1050 (0.2)	0.028	195 (0.4)	162 (0.3)	0.011
Smoking status						
Never smoker	39,738 (81.0)	291,628 (62.9)		39,735 (81.0)	40,063 (81.7)	
Previous smoker	2112 (4.3)	41,404 (8.9)	0.228	2112 (4.3)	2064 (4.2)	0.005
Current smoker	5189 (10.6)	112,137 (24.2)	0.442	5189 (10.6)	4951 (10.1)	0.016
Unknown	2003 (4.1)	18,694 (4.0)	0.003	2003 (4.1)	1961 (4.0)	0.004
Alcohol consumption (frequency)						
No drink	35,365 (72.1)	250,644 (54.0)		35,362 (72.1)	35,364 (72.1)	
2–3 per a month	5535 (11.3)	71,024 (15.3)	0.127	5535 (11.3)	5671 (11.6)	0.009
1–2 per a week	4054 (8.3)	78,927 (17.0)	0.318	4054 (8.3)	4053 (8.3)	< 0.001
3–4 per a week	1508 (3.1)	33,906 (7.3)	0.245	1508 (3.1)	1410 (2.9)	0.012
Almost everyday drink	1384 (2.8)	20,995 (4.5)	0.103	1384 (2.8)	1388 (2.8)	< 0.001
Unknown	1196 (2.4)	8367 (1.8)	0.041	1196 (2.4)	1153 (2.4)	0.006
Exercise frequency						
No exercise	31,266 (63.8)	254,500 (54.9)		31,264 (63.8)	30,689 (62.6)	
1–2 per a week	8461 (17.3)	109,692 (23.6)	0.127	8460 (17.3)	8635 (17.6)	0.009
3–4 per a week	3543 (7.2)	43,539 (9.4)	0.318	3543 (7.2)	3801 (7.8)	0.020
5–6 per a week	1132 (2.3)	12,043 (2.6)	0.245	1132 (2.3)	1163 (2.4)	0.004
Almost everyday	3372 (6.9)	31,328 (6.8)	0.103	3372 (6.9)	3427 (7.0)	0.004
Unknown	1268 (2.6)	12,761 (2.8)	0.041	1268 (2.6)	1324 (2.7)	0.007
Charlson comorbidity index	1.5 (1.9)	1.3 (1.6)	0.136	1.5 (1.9)	1.5 (1.8)	0.019
Myocardial infarction	509 (1.0)	3925 (0.8)	0.019	509 (1.0)	460 (0.9)	0.010
Congestive heart failure	1814 (3.7)	12,412 (2.7)	0.054	1813 (3.7)	1688 (3.4)	0.014
Peripheral vascular disease	2183 (4.5)	15,702 (3.4)	0.052	2182 (4.4)	2026 (4.1)	0.015
Cerebrovascular disease	2608 (5.3)	19,873 (4.3)	0.054	2607 (5.3)	2526 (5.2)	0.007
Dementia	271 (0.6)	1757 (0.4)	0.023	271 (0.6)	261 (0.5)	0.003
Chronic pulmonary disease	12,497 (25.5)	104,209 (22.5)	0.069	12,495 (25.5)	12,513 (25.5)	< 0.001
Rheumatic disease	3631 (7.4)	24,377 (5.3)	0.072	3630 (7.4)	3537 (7.2)	0.007
Peptic ulcer disease	16,890 (34.4)	139,279 (30.0)	0.093	16,888 (34.4)	16,696 (34.0)	0.008
Mild liver disease	7800 (15.9)	76,669 (16.5)	0.017	7798 (15.9)	7813 (15.9)	< 0.001
Diabetes without chronic complication	2923 (6.0)	26,396 (5.7)	0.011	2923 (6.0)	2949 (6.0)	0.002
Diabetes with chronic complication	2038 (4.2)	16,010 (3.5)	0.035	2038 (4.2)	1920 (3.9)	0.012
Hemiplegia or paraplegia	276 (0.6)	1901 (0.4)	0.020	276 (0.6)	257 (0.5)	0.005
Renal disease	595 (1.2)	1971 (0.4)	0.072	592 (1.2)	455 (0.9)	0.026
vAny malignancy	6889 (14.0)	50,533 (10.9)	0.091	6888 (14.0)	6784 (13.8)	0.006
Moderate or severe liver disease	277 (0.6)	1341 (0.3)	0.037	275 (0.6)	242 (0.5)	0.009
Metastatic solid tumour	454 (0.9)	2075 (0.4)	0.050	453 (0.9)	401 (0.8)	0.011
AIDS/HIV	0 (0.0)	1 (0.0)	< 0.001	0 (0.0)	0 (0.0)	< 0.001

Presented as mean value with standard deviation or number with percentage

PS, propensity score; ASD, absolute value of standardized mean difference; AIDS, acquired immune deficiency syndrome; HIV, human immunodeficiency virus

### Mortality due to infection and history of anemia

**Fig. 2:**
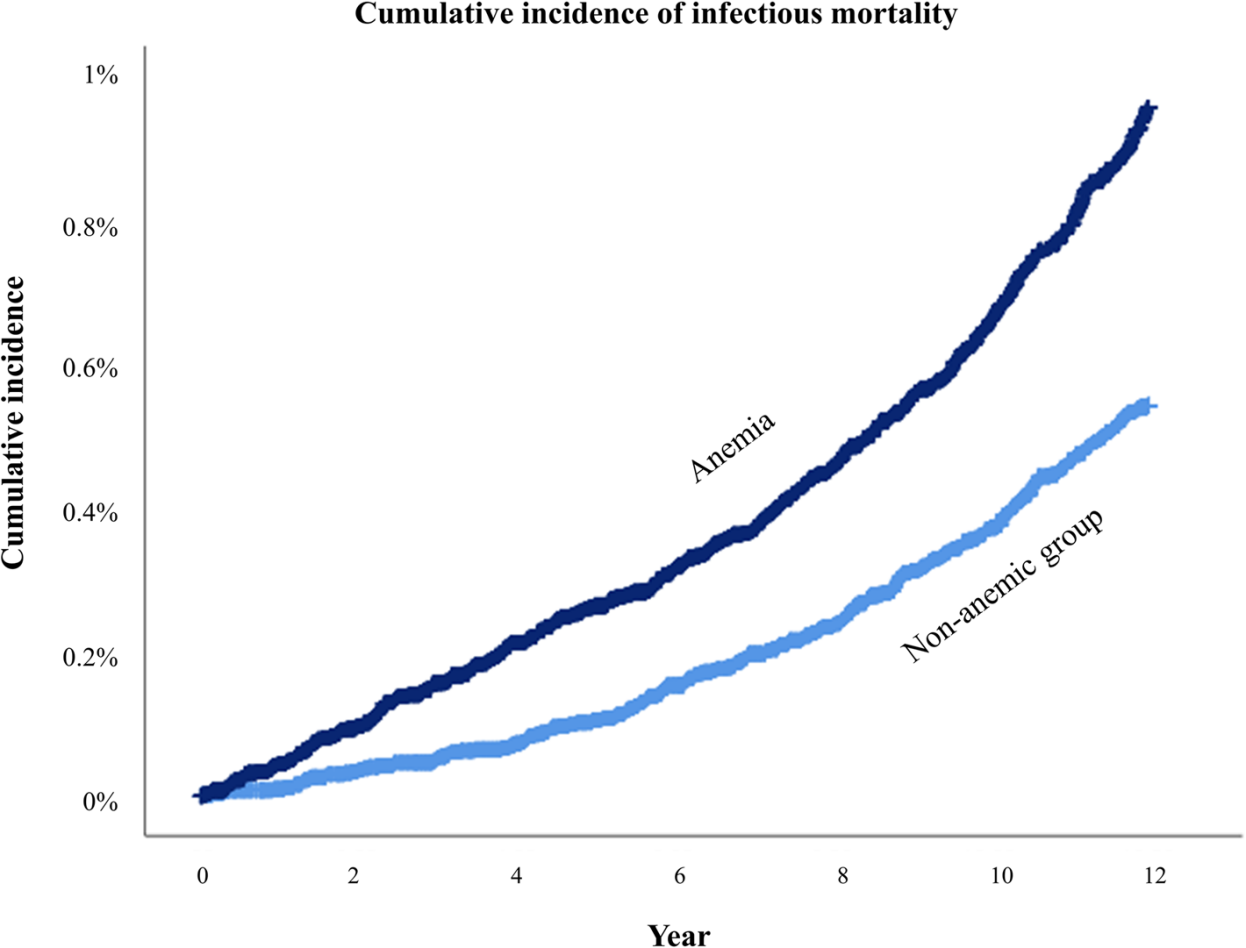
Cumulative incidence of mortality due to infection between the anemic group and non-anemic group in the propensity score matched cohort

Table 2 shows the results of the analysis related to mortality due to infection during 2004–2015 before and after PS-matching. After PS-matching, the mortality due to infection in the anemic group was higher (0.9%; 437/49,039) than that in the non-anemic group (0.5%; 256/49,039). In the Cox regression analysis, the risk of mortality in the anemic group was 1.77-fold higher (HR: 1.77, 95% CI: 1.52 to 2.60; *P* < 0.001) than that in the non-anemic group. The incidence of mortality due to infection in the two groups had a similar trend, as shown in Fig.2. Table S3 shows the results of the analysis related to mortality due to infection during 2005–2015 before and after PS-matching. In the Cox regression analysis, the risk of mortality due to infection in the anemic group was 1.77-fold higher (HR: 1.77, 95% CI: 1.47 to 2.01; *P* < 0.001) than that in the non-anemic group.

**Table 2:** Mortality due to infection during 2004–2015 before and after PS matching

Variable	Infectious mortality (n, %)	Cox regression	*P*-value
		HR (95% CI)	
Before PS matching			
Non-anemic group	2008/463,863 (0.4)	1	
Anemia group	437/49,042 (0.9)	2.14 (1.93, 2.37)	< 0.001
After PS matching			
Non-anemic group	256/49,039 (0.5)	1	
Anemia group	437/49,039 (0.9)	1.77 (1.52, 2.06)	< 0.001

PS, propensity score; HR, hazard ratio; CI, confidence interval

Table 3 shows the results of the multivariable Cox regression model for mortality due to infection in the entire cohort. The anemic group had a 1.47-fold higher mortality due to infection than the non-anemic group (HR: 1.47, 95% CI: 1.33 to 1.64; *P* < 0.001; Model 1). In the subgroup analysis, the mild and moderate historical anemia groups had 1.38-fold (HR: 1.38, 95% CI: 1.23 to 1.55; *P* < 0.001; Model 2) and 2.02-fold (HR: 2.02, 95% CI: 1.62 to 2.50; *P* < 0.001; Model 2) higher mortality due to infection than the non-anemic group, respectively. However, the severe anemic group did not show a significant difference in mortality due to infection compared to that of the non-anemic group (*P* = 0.448). The C-index of the multivariable model was 0.85 (95% CI: 0.84 to 0.86). The Table 4 shows the results of subgroup analysis stratified by sex. The male anemic group (*n* = 277,846) had a 1.57-fold higher mortality due to infection than the male non-anemic group (HR: 1.57, 95% CI: 1.37 to 1.83; *P* < 0.001). The female anemic group (*n* = 235,059) had a 1.36-fold higher mortality due to infection than the female non-anemic group (HR: 1.36, 95% CI: 1.15 to 1.61; *P* < 0.001).

**Table 3:** Multivariable Cox regression model for mortality due to infection during 2004–2015 in entire cohort

Variable	Multivariable model	*P*-value
	HR (95% CI)	
Anemia (vs non-anemic group, model 1)	1.47 (1.33, 1.64)	< 0.001
Subgroup analysis (model 2)		
Non-anemic group	1	
Mild anemia (*n* = 36,383, 7.1%)	1.38 (1.23, 1.55)	< 0.001
Moderate anemia (*n* = 11,787, 2.3%)	2.02 (1.62, 2.50)	< 0.001
Severe anemia (*n* = 872, 0.2%)	1.55 (0.50, 4.82)	0.448
Age, year	1.16 (1.15, 1.37)	< 0.001
Sex, male	2.16 (1.96, 2.39)	< 0.001
Residence at diagnosis of sepsis		
Seoul (Capital city)	1	
Other metropolitan city	1.18 (1.02, 1.37)	0.027
Other area	1.24 (1.08, 1.41)	0.002
Body mass index, kg/m^2^		
18.5–24.9 (normal)	1	
Below 18.5 (Underweight)	2.33 (2.04, 2.66)	< 0.001
25.0–29.9 (Overweight)	0.74 (0.67, 0.81)	< 0.001
Above 30.0 (Obese)	1.07 (0.83, 1.37)	0.625
Unknwon	0.33 (0.05, 2.36)	0.270
Annual income level		
0–20%	1	
20–40%	0.91 (0.80, 1.03)	0.143
40–60%	0.84 (0.74, 0.96)	0.008
60–80%	0.74 (0.65, 0.83)	< 0.001
80–100%	0.65 (0.58, 0.73)	< 0.001
Underlying disability		
Mild to moderate	1.19 (0.88, 1.59)	0.258
Severe	1.63 (1.12, 2.37)	0.011
Smoking status		
Never smoker	1	
Previous smoker	1.34 (1.16, 1.54)	< 0.001
Current smoker	1.50 (1.35, 1.67)	< 0.001
Unknown	1.08 (0.83, 1.39)	0.580
Alcohol consumption (frequency)		
No drink	1	
2–3 per a month	0.73 (0.63, 0.85)	< 0.001
1–2 per a week	0.91 (0.80, 1.05)	0.199
3–4 per a week	0.82 (0.69, 0.99)	0.034
Almost everyday drink	1.01 (0.87, 1.18)	0.867
Unknown	0.93 (0.66, 1.31)	0.688
Exercise frequency		
No exercise	1	
1–2 per a week	0.81 (0.71, 0.92)	0.001
3–4 per a week	0.74 (0.61, 0.89)	0.002
5–6 per a week	0.72 (0.52, 0.99)	0.044
Almost everyday	0.79 (0.68, 0.92)	0.002
Unknown	1.22 (0.95, 1.58)	0.128
Charlson comorbidity index, 1 point (model 2)	1.13 (1.11, 1.15)	< 0.001
Myocardial infarction	1.19 (0.91, 1.55)	0.198
Congestive heart failure	1.18 (1.01, 1.3)	0.038
Peripheral vascular disease	1.22 (1.06, 1.40)	0.007
Cerebrovascular disease	1.29 (1.13, 1.47)	< 0.001
Dementia	1.48 (1.07, 2.04)	0.018
Chronic pulmonary disease	1.26 (1.15, 1.37)	< 0.001
Rheumatic disease	1.29 (1.13, 1.47)	< 0.001
Peptic ulcer disease	0.91 (0.83, 0.99)	0.028
Mild liver disease	1.24 (1.13, 1.37)	< 0.001
Diabetes without chronic complication	1.25 (1.10, 1.43)	0.001
Diabetes with chronic complication	1.36 (1.16, 1.59)	< 0.001
Hemiplegia or paraplegia	1.13 (0.79, 1.64)	0.503
Renal disease	0.92 (0.60, 1.40)	0.692
Any malignancy	1.15 (1.03, 1.28)	0.014
Moderate or severe liver disease	2.30 (1.55, 3.43)	< 0.001
Metastatic solid tumour	0.66 (0.37, 1.18)	0.162
AIDS/HIV	0.00 (0.00-)	0.997

HR, hazard ratio; CI, confidence interval; AIDS, acquired immune deficiency syndrome; HIV, human immunodeficiency virus

**Table 4:** Subgroup analysis according to sex

Variable	Multivariable model	*P*-value
	HR (95% CI)	
Male (n = 277,846, event = 1571)		
Anemia group (vs non-anemic group)	1.57 (1.37, 1.83)	< 0.001
Subgroup analysis		
Non-anemic group	1	
Mild historical anemia	1.49 (1.28, 1.72)	< 0.001
Moderate historica anemia	2.49 (1.6, 3.51)	< 0.001
Severe historica anemia	2.06 (0.51, 8.26)	0.308
Female (235,059, event = 874)		
Anemia group (vs non-anemic group)	1.36 (1.15, 1.61)	< 0.001
Subgroup analysis		
Non-anemic group	1	
Mild historica anemia	1.23 (1.01, 1.49)	0.038
Moderate historica anemia	1.84 (1.39, 2.43)	< 0.001
Severe historica anemia	0.99 (0.14, 7.07)	0.994

HR, hazard ratio; CI, confidence interval

## Discussion

This population-based cohort study showed that history of anemia was independently associated with increased infectious mortality in South Korea. Interestingly, in the subgroup analyses, it was more evident in the mild and moderate anemic groups than the severe anemic group. We hypothesized that patients with a history of anemia have a higher risk of death due to infection during the long-term follow-up period. Considering that the prevalence of both anemia and infection was high in the low-income countries [24], our result can be applicable in the low-income countries, significantly.

Anemia, iron deficiency, and infections have been reported as three major causes of mortality and morbidity during childhood worldwide [25]. These three conditions have a close relationship and may interact. For example, iron deficiency leads to anemia and increases susceptibility to infection by immunosuppression [26]. In contrast, iron replacement in patients with IDA can also increase the incidence of infection because iron is a necessary nutrient for many pathogens [27]. The bacteriostatic effects of iron-binding proteins were first discovered in 1944 by Schade and Caroline [28]. These effects are mediated by hepcidin, which is the master regulator of iron hemostasis [29]. Hepcidin induction during infection causes depletion of extracellular iron, which acts as a defense mechanism against infection by withholding iron from invading pathogens. However, by promoting iron sequestration in macrophages, hepcidin may be detrimental to the cellular defense system against intracellular infections [30]. Independent of the role of hepcidin, several other cytokines such as tumor necrosis factor alpha, interferon gamma, interleukin-1, and interleukin-6 also modulate iron metabolism and the iron-withholding defense during infection [31]. Therefore, the patients with IDA in the anemic groups in our cohort might be more susceptible to mortality due infection because of an impaired immune defense system.

However, a previous study reported that > 50% of anemia was developed due to other reasons such as deficiency of micronutrients (vitamin A, folate, vitamin D, and vitamin B12) rather than IDA alone [32]. The malnutrition is known to increase both frequency of infection and long-term mortality due to infection [33]. Our study suggested that the individuals with a history of anemia might have nutritional deficiency compared to non-anemic individuals in South Korea, which results in a higher risk of infection. Deficiency of one of the micronutrients, vitamin D, is common in South Korea [34], and this may aid in the development of anemia [35].

History of anemia might also affect the prognosis of individuals after infection. Anemia has been reported to be common among hospitalized patients with acquired pneumonia, and it was associated independently with higher 90-day mortality [36]. Moreover, for surgical patients, the incidence of preoperative anemia has been reported to be as high as 33.9%, and it was associated with a higher risk of perioperative infection and mortality [37]. Anemia is also common in patients with sepsis, which is the most severe condition among infections, and has also been reported to increase mortality among these patients [38]. Since our study focused on mortality due to infection in an adult population, our results not only reflect the susceptibility of infection in patients with a history of anemia, but also the poorer prognosis of these patients after hospitalization for treatment of infection.

A recent study has reported that the comorbid status of anemia is associated with an enhanced risk of severe COVID-19 infection, with an odds ratio of 2.44 (95% CI: 1.75 to 3.40) [39]. In the circulation system, hemoglobin delivers oxygen to major organs in the body. When the blood hemoglobin level is low, the oxygen delivery to major organs will be disrupted, leading to hypoxia, which will eventually result in multiple organ dysfunction among infected patients such as those with COVID-19 [40]. Our results suggest that infected individuals with a history of anemia will have a higher incidence of infection and risk of increased mortality. This can also be applied to patients with COVID-19 in the current COVID-19 pandemic.

The results of the subgroup analysis are notable: the severe anemic group (hemoglobin < 8 g/dL) was not associated with mortality due to infection; whereas, the mild and moderate anemic groups were associated with a higher risk of mortality due to infection. Anemia has been reported to increase hospitalization and mortality in older adults [41], and it might be more evident for patients with a history of severe anemia. IDA is well known as a major risk factor for the development of cardiovascular disease [42] because low hemoglobin levels have adverse effects on myocardial and large arterial remodeling [43]. A previous study reported that a history of severe anemia was associated with a higher risk of death and myocardial ischemia [44]. Thus, it is possible that the impact of history of severe anemia was higher for other mortalities, such as cardiovascular mortality than that due to an infection in our study; therefore, the impact of history of severe anemia should be considered carefully. However, the reason for the non-significant association between history of severe anemia and 12-year mortality is unclear, and further studies are warranted in this regard.

Our study has some limitations. First, we did not distinguish the type of anemia in detail because we only used the measured hemoglobin level in the standardized medical examination. Furthermore, information on treatment of the anemia was not evaluated during follow-up period (2004–2015) in this study, and it might affect the result of this study. Second, PS-matching, and multivariable adjustment only adjusted for known confounders, and there might be unmeasured confounders that may have affected the results in this study. Third, we used ICD-10 codes to define comorbid status and calculate the Charlson comorbidity index, but actual underlying diseases might differ with the registered ICD-10 codes in this study. For example, if individuals with diabetes mellitus did not visit outpatient clinic due to mild symptom or poor accessibility to healthcare utilization, they were not registered as diabetes mellitus in the NHIS database in this study. Fourth, we did not exclude women who were pregnant during 2002–2003 in this study. However, since our inclusion criteria were adults ≥40 years old, we believe that the impact of pregnancy might be limited in this study. Fifth, lifestyle information was collected using a questionnaire, and there might be a selection bias due to non-response to the surveys in the standardized medical examination [45]. Lastly, since the data was collected retrospectively in this study, the validity and credibility might be limited.

## Conclusions

We showed that a history of anemia was associated with increased mortality due to infection among the adult South Korean population, and this was more evident in patients with mild to moderate anemia than those with severe anemia. However, considering the limitations of this study, further studies are warranted to confirm our findings.

## Supplementary Information

**Additional file 1.**

**Additional file 2.**

**Additional file 3.**

## Data Availability

The data that support the findings of this study are available from National Health Insurance System, but restrictions apply to the availability of these data, which were used under license for the current study and so are not publicly available. Data are, however, available from the authors upon reasonable request and with permission from the National Health Insurance System (https://nhiss.nhis.or.kr/bd/ab/bdaba000eng.do). If someone wants to request the data from this study, please contact to corresponding author (songoficu@outlook.kr).

## Abbreviations

COVID-19: coronavirus disease-2019
IDA: iron deficiency anemia
NHIS: national health insurance service
NHIS-HEALS: NHIS-National Health Screening Cohort
ICD: International Classification of Diseases
BMI: body mass index
WHO: World Health Organization
PS: propensity score
ASD: absolute value of the standardized mean difference
HR: hazard ratio
CI: confidence interval
