# Supplementary material for: History of anemia and long-term mortality due to infection: a cohort study with 12 years follow-up in South Korea
Source: BMC Infect Dis. 2021 Jul 11;21:674. doi: 10.1186/s12879-021-06377-0 (PMC8272955; doi:10.1186/s12879-021-06377-0)
Supplement: Supplementary file 1 — Additional file 1. [file 12879_2021_6377_MOESM1_ESM.docx]

**History of anemia and Long-term mortality due to infection: A Cohort Study with 12 years Follow-up in South Korea**

Subtitle: Anemia and Infection

Tak Kyu Oh^1^, Kyung-Ho Song^2^, and In-Ae Song^1^

^1^Department of Anesthesiology and Pain Medicine, Seoul National University Bundang Hospital, Seongnam, Korea

^2^Department of Internal Medicine, Seoul National University Bundang Hospital, Seongnam, Korea

Corresponding Author: In-Ae Song

Department of Anesthesiology and Pain Medicine, Seoul National University Bundang Hospital, 166 Gumi-ro, Bundang-gu, Seongnam 463-707, Korea

Tel: +82-31-787-7499, Fax: +82-31-787-4063, Email: songoficu@outlook.kr

**Table S1. ICD-10 codes related to an infection**

A00-A09 Intestinal infectious diseases

A15-A19 Tuberculosis

A20-A28 Certain zoonotic bacterial diseases

A30-A49 Other bacterial diseases

A50-A64 Infections with a predominantly sexual mode of transmission

A70-A74 Other diseases caused by chlamydia

A75-A79 Rickettsioses

A92-A99 Arthropod-borne viral fevers and viral hemorrhagic fevers

B00 Herpes viral [herpes simplex] infections

B01 Varicella [chickenpox]

B02 Zoster [herpes zoster]

B05 Measles

B06 Rubella [German measles]

B15-B19 Viral hepatitis

B25-B34 Other viral diseases

B37 Candidiasis

B38 Coccidioidomycosis

B39 Histoplasmosis

B40 Blastomycosis

B44 Aspergillosis

B49 Unspecified mycosis

B50-B64 Protozoal diseases

B99 Other infectious diseases

G00 Bacterial meningitis, not elsewhere classified

G01 Meningitis in bacterial diseases classified elsewhere

G02 Meningitis in other infectious and parasitic diseases classified elsewhere

G04 Encephalitis, myelitis and encephalomyelitis

G05 Encephalitis, myelitis and encephalomyelitis in diseases classified elsewhere

G06 Intracranial and intraspinal abscess and granuloma

G07 Intracranial and intraspinal abscess and granuloma in diseases classified elsewhere

G08 Intracranial and intraspinal phlebitis and thrombophlebitis

H05.0 Acute inflammation of orbit

H44.0 Purulent endophthalmitis

H60.2 Malignant otitis externa

H70.0 Acute mastoiditis

I30.1 Infective pericarditis

I33.0 Acute and subacute infective endocarditis

J01 Acute sinusitis

J02 Acute pharyngitis

J03 Acute tonsillitis

J04 Acute laryngitis and tracheitis

J06 Acute upper respiratory infections of multiple and unspecified sites

J09-J18 Influenza and pneumonia

J20-22 Other acute lower respiratory infections

J36 Peritonsillar abscess

I40.0 Infective myocarditis

J39.0 Retropharyngeal and parapharyngeal abscess

J39.1 Other abscess of pharynx

J44.0 Chronic obstructive pulmonary disease with acute lower respiratory infection

J47.0 Bronchiectasis

J85-J86 Suppurative and necrotic conditions of lower respiratory tract

J98.50 Mediastinitis

K10.2 Inflammatory conditions of jaws

K11.3 Abscess of salivary gland

K12.2 Cellulitis and abscess of mouth

K35 Acute appendicitis

K57.0 Diverticular disease of small intestine with perforation and abscess

K57.2 Diverticular disease of large intestine with perforation and abscess

K57.4 Diverticular disease of both small and large intestine with perforation and abscess

K57.8 Diverticular disease of intestine, part unspecified, with perforation and abscess

K61 Abscess of anal and rectal regions

K63.0 Abscess of intestine

K63.1 Perforation of intestine (nontraumatic)

K65 Peritonitis

K75.0 Abscess of liver

K75.1 Phlebitis of portal vein

K81.0 Acute cholecystitis

K83.0 Cholangitis

L00 Staphylococcal scalded skin syndrome

L01 Impetigo

L02 Cutaneous abscess, furuncle and carbuncle

L03 Cellulitis

L04 Acute lymphadenitis

L05 Pilonidal cyst

L08 Other local infections of skin and subcutaneous tissue

L88 Pyoderma gangrenosum

M00 Pyogenic arthritis

M01 Direct infections of joint in infectious and parasitic diseases classified elsewhere

M46.2 Osteomyelitis of vertebra

M46.3 Infection of intervertebral disc (pyogenic)

M46.5 Other infective spondylopathies

M60.0 Infective myositis

M72.6 Necrotizing fasciitis

M86 Osteomyelitis

N10 Acute tubulo-interstitial nephritis

N30.0 Acute cystitis

N30.8 Other cystitis

N30.9 Cystitis, unspecified

N39.0 Urinary tract infection, site not specified

N41.0 Acute prostatitis

N41.2 Abscess of prostate

N41.3 Prostatocystitis

N41.8 Other inflammatory diseases of prostate

N45 Orchitis and epididymitis

N70-77 Inflammatory diseases of female pelvic organs

O03.0 Spontaneous abortion : incomplete, complicated by genital tract and pelvic infection

O03.5 Spontaneous abortion : complete or unspecified, complicated by genital tract and pelvic infection

O04.0 Medical abortion : incomplete, complicated by genital tract and pelvic infection

O05.0 Other abortion : incomplete, complicated by genital tract and pelvic infection

O06.0 Unspecified abortion : incomplete, complicated by genital tract and pelvic infection

O07.0 Failed medical abortion, complicated by genital tract and pelvic infection

O08.0 Genital tract and pelvic infection following abortion and ectopic and molar pregnancy

O23 Infections of genitourinary tract in pregnancy

O41.1 Infection of amniotic sac and membranes

O75.3 Other infection during labour

O85 Puerperal sepsis

O86 Other puerperal infections

R57.2 Septic shock

R65.0 Systemic Inflammatory Response Syndrome of infectious origin without organ failure

R65.1 Systemic Inflammatory Response Syndrome of infectious origin with organ failure

T79.3 Post-traumatic wound infection, not elsewhere classified

T82.x - T85.x Infection and inflammatory reaction due to other cardiac and vascular devices, implants and grafts
