# Supplementary material for: History of anemia and long-term mortality due to infection: a cohort study with 12 years follow-up in South Korea
Source: BMC Infect Dis. 2021 Jul 11;21:674. doi: 10.1186/s12879-021-06377-0 (PMC8272955; doi:10.1186/s12879-021-06377-0)
Supplement: Supplementary file 3 — Additional file 3. [file 12879_2021_6377_MOESM3_ESM.docx]

**History of anemia and Long-term mortality due to infection: A Cohort Study with 12 years Follow-up in South Korea**

Subtitle: Anemia and Infection

Tak Kyu Oh^1^, Kyung-Ho Song^2^, and In-Ae Song^1^

^1^Department of Anesthesiology and Pain Medicine, Seoul National University Bundang Hospital, Seongnam, Korea

^2^Department of Internal Medicine, Seoul National University Bundang Hospital, Seongnam, Korea

Corresponding Author: In-Ae Song

Department of Anesthesiology and Pain Medicine, Seoul National University Bundang Hospital, 166 Gumi-ro, Bundang-gu, Seongnam 463-707, Korea

Tel: +82-31-787-7499, Fax: +82-31-787-4063, Email: songoficu@outlook.kr

Table S3. Infectious mortality during 2005-2015 before and after PS matching

| Variable | | Infectious mortality (n, %) | Cox regression | *P*-value |
| --- | --- | --- | --- | --- |
|  |  |  | HR (95% CI) |  |
| Before PS matching | |  |  |  |
|  | Non-anemic group | 1,961/463,863 (0.4) | 1 |  |
|  | Anemia group | 415/49,042 (0.8) | 2.07 (1.87, 2.31) | <0.001 |
| After PS matching | |  |  |  |
|  | Non-anemic group | 251/49,039 (0.5) | 1 |  |
|  | Anemia group | 415/49,039 (0.8) | 1.71 (1.47, 2.01) | <0.001 |

HR, hazard ratio; CI, confidence interval; PS, propensity score
